# The miRNA variants *MIR196A2* (rs11614913) and *MIR423* (rs6505162) contribute to an increase in the risk of myocardial infarction

**DOI:** 10.1002/mgg3.2323

**Published:** 2023-11-27

**Authors:** Muhammad Uzair, Taqweem Ul Haq, Sajjad Ali, Manzar Hussain, Fazal Jalil, Yasir Ali, Aftab Ali Shah

**Affiliations:** ^1^ Department of Biotechnology, Faculty of Biological Sciences University of Malakand Chakdara Pakistan; ^2^ Department of Biotechnology Abdul Wali Khan University Mardan (AWKUM) Mardan Pakistan; ^3^ School of Biomedical Sciences The Chinese University of Hong Kong Hong Kong Hong Kong

**Keywords:** genotyping, myocardial infarction, Pakistani population, SNP, T‐ARMS‐PCR

## Abstract

**Introduction:**

MicroRNAs (miRNAs) are small, single‐stranded RNA molecules that negatively regulate gene expression and play a key role in the pathogenesis of human diseases. Recent studies have suggested that miRNAs contribute to cardiovascular diseases (CVDs). However, the association between single‐nucleotide polymorphisms (SNPs) in miRNAs and myocardial infarction (MI) remains in infancy.

**Aim:**

The current study was designed to find out the association of SNPs in *MIR196A2* and *MIR423* (rs11614913 and rs6505162, respectively).

**Methods:**

Using Tetra‐Primer Amplification Refractory Mutation System‐Polymerase Chain Reaction (T‐ARMS PCR) in 400 cases (MI patients) and 336 healthy controls. Using different inheritance models (co‐dominant, homozygous dominant, homozygous recessive, and additive models), the association of these SNPs was genotyped with MI risk.

**Results:**

For variant rs11614913, significant distribution of the genotypes among the cases and controls was determined by co‐dominant [*χ* ^2^ = 29.19, 2; *p* value < 0.0001], dominant (C/C vs. C/T + T/T) [OR = 0.45 (0.34 to 0.61); *p* < 0.0001], recessive (T/T vs. C/T + C/C) [OR = 1.009 (0.63 to 1.63); *p‐value* *p* value > 0.999], and additive models [OR = 0.65 (0.52 to 0.80); *p* value = 0.0001]. Similarly, a significant association of rs6505162 was determined by co‐dominant [*χ* ^2^ = 24.29, 2; *p* value < 0.0001], dominant (C/C vs. A/C+ A/A) [OR = 0.44 (0.32 to 0.61); *p* value < 0.0001], recessive (A/A vs. A/C + C/C) [OR = 1.29 (0.85 to 1.98); *p* value = 0.28], and additive models [OR = 0.65 (0.52 to 0.81); *p* value = 0.0001].

**Conclusion:**

Therefore, the current study showed that both variants rs11614913 and rs6505162 are significantly associated with MI in the Pakistani population.

## Graphical abstract

Genotyping of selected miRNA genes in myocardial infraction patients

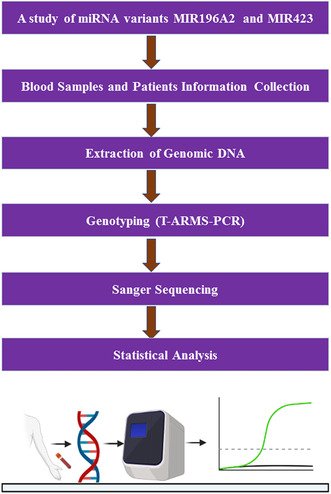

## 1 INTRODUCTION

Cardiovascular diseases (CVDs) are a group of pathological conditions affecting the heart and blood vessels (Mendis et al., 2011).CVDs are the major cause of death in developing and developed countries (Zhou et al., 2018). From 1990 to 2019, the number of prevalent cases of cardiovascular diseases doubled from 271 million to 523 million, and the number of CVD deaths increased from 12.1 million to 18.6 million (Roth et al., 2020). South Asia comprises about a quarter of the world's population, but they account for more than half of all cardiovascular mortality worldwide (Gupta et al., 2020). In 2019, Asia accounted for 58% of the 18.6 million deaths due to CVDs worldwide (Zhao, 2021). The most prevalent kind of CVD is myocardial infarction (MI) (Khalid et al., 2020). MI is also known as heart attack and is usually occurred due to limited or no blood flow to the heart which results in necrosis of the heart muscles. MI is classified as either ST‐segment elevation myocardial infarction (STEMI) or non‐ST‐segment elevation myocardial infarction (NSTEMI) based on the presence or absence of ST‐segment elevation in the electrocardiography (ECG).

MiRNAs are a class of short single‐stranded non‐coding RNA molecules that negatively regulate gene expression in the post‐transcriptional stage through interaction with target messenger RNAs (mRNAs), thus leading to either translational inhibition or gene silencing. MiRNAs are divided into several groups based on their sequence location in the genome (Kamanu et al., 2013). Many miRNAs are grouped into polycistronic clusters, resulting in a large number of miRNAs from a single main transcript (Concepcion et al., 2012). MiRNA is thought to regulate about 60 percent of human protein‐coding genes by the post‐transcriptional process (Colpaert & Calore, 2019). In the human genome, there are now 2500 miRNAs annotated (Leptidis et al., 2013). Recent research has shown that the aberrant expression of miRNA plays a pivotal role in the pathogenesis of MI (Vickers et al., 2014). The understanding of the loci and genetic variations that contribute to the pathophysiology of MI has recently improved because of genome‐wide association studies (GWAS) (Ramezanpour et al., 2020). The miRNAs i.e. miR‐133 and miR‐1 are highly expressed in the cardiac cells play an important role in the inhibition of heart cell proliferation and differentiation, respectively (Çakmak & Demir, 2020). Furthermore, several miRNAs i.e. miR‐195, miR‐1, miR‐590, miR‐208a, miR‐126, and miR‐499 play an important role in the development of the cardiovascular system (Sayed & Abdellatif, 2011). STEMI was shown to have higher levels of miRNA expression than NSTEMI. However, in the STEMI group, miR‐145 was shown to be lower than in the NSTEMI group (Navickas et al., 2016). Another study shows that gene expression is negatively regulated by miRNA that operates on mRNA (Catalanotto et al., 2016).

Mismatching of miRNA–mRNA binding may have a role in the onset of a variety of human diseases including MI. This mismatching could be caused by a variation in the miRNA target region or the miRNA genes (Buraczynska et al., 2014; Jha et al., 2019; Nariman‐Saleh‐Fam et al., 2017; Parvin et al., 2019; Rong et al., 2017; Wu et al., 2017). SNPs in miRNA could have an impact on them at several stages, including miRNA maturation, the gene silencing machinery, and the structure and expression of mature miRNA. Furthermore, SNPs might impact base pairing at the target location, which can affect miRNA‐regulated gene expression, thereby raising disease risk, such as MI.

Previous studies have shown that polymorphisms in miR‐146a (Xiong et al., 2014), miR‐149 (Ding et al., 2013), miR‐196a2 (Zhi et al., 2012; Zhou et al., 2010), miR‐499 (Liu et al., 2014; Zhi et al., 2012), and miR‐423 (He et al., 2022) are involved in the progression of CVDs including ischemic stroke, CAD, and MI (Chen et al., 2014). The rs2910164 in miRNA‐146a was found to be linked with the risk of an acute coronary syndrome (ACS) and CAD (Huang et al., 2015). Cardiac miRNAs (miR‐499, miR133a, miR‐1, and miR‐208a/b), which are widely expressed in the myocardium, can have tissue‐specific expression patterns. There is increasing evidence that these miRNAs are involved in heart development and some cardiovascular disorders, such as MI (Xue et al., 2017). This study aimed to investigate the correlation between two SNPs, rs11614913 in microRNA‐196a and rs6505162 in microRNA‐423, and their association with MI occurrence. The rs11614913 polymorphism in the mature region of miR‐196a‐2 and has been extensively studied and is associated with the risk and prognosis of various diseases (Xiong et al., 2014). The rs6505162 C > A polymorphism is located in the premir‐423 region and has been reported to stimulate the expression of mature mir‐423 (Su et al., 2015). The previous studies have reported that miR‐423 regulates various vascular diseases, such as atherosclerosis (Zeng et al., 2018), myocardial infarction (Bauters et al., 2013), diabetic vascular complications (Hirota et al., 2015), and lymphoma (Ayoubian et al., 2019).

## 2 MATERIALS AND METHODS

### 2.1 Ethical standard, samples collection, and extraction of genomic DNA

The study received approval from the University of Malakand Advanced Study and Research Board Notification No. UOM/Admin/2021/1560, following the guidelines of the Helsinki Declaration. Informed consent was obtained from healthy individuals and patients. The sample consisted of 400 patients diagnosed with acute myocardial infarction (AMI) based on the criteria outlined in the Third Universal Definition of Myocardial Infarction (Ayoubian et al., 2019). The participants enrolled in this study met the predetermined inclusion criteria. Cardiac markers, such as troponin, are clinically detectable once their concentrations surpass the 99th percentile threshold of the upper limit within the normal reference range. At least one of the following clinical signs should be present in addition to this finding: myocardial ischemia symptoms and then improvement after receiving blood through the coronary arteries, pain in the chest that lasts for more than 30 min may radiate to the extremities, neck, jaw, and back and may be accompanied by nausea, vomiting, dyspnea, and diaphoresis. Furthermore, diagnostic indicators such as pathological Q‐waves, significant alterations in ST‐segment elevation or depression, and changes in T‐wave morphology on an electrocardiogram (ECG) may be observed. Additionally, imaging assessments may reveal recent myocardial damage or the emergence of new segmental wall motion abnormalities. A sample of 336 healthy individuals who had no prior history of cardiovascular events such as heart attack or other diseases were selected as the control group. The present study excluded patients with a history of non‐coronary cardiac disorders, uncontrolled blood pressure, diabetes, chest pain resulting from trauma or drug use, prior coronary bypass surgery, and other contraindications such as hepatic or nephritic conditions and renal disease. The study documented demographic variables, such as age, gender, and medical history, for all participants. Both AMI patients and healthy individuals had 5 cubic centimeters (CC) of whole blood collected using ethylenediaminetetraacetic acid (EDTA) tubes. The genomic DNA extraction from blood samples was done through the phenol/chloroform procedure. The DNA obtained was dissolved in a solution of 50 microliters of nuclease‐free water and stored at 4 degrees Celsius.

### 2.2 Selection of SNPs

The website miRbase (http://www.mir.org) has data on the locations of 1917 precursor and 2654 mature human miRNAs. We used miRbase to find information on the SNPs being studied. The details on the chromosomal site and alleles of rs11614913 T/C and rs6505162 C/A were obtained from the dbSNP database.

### 2.3 Genotyping of *MIR196A2* (rs11614913)

Table 1 shows detailed information about the studied SNPs (rs11614913 & rs6505162), their approved gene names, mature miRNAs, location (coordinates) on their respective chromosomes, and coded alleles, another allele, and minor allele frequencies, as well as their approved gene names. Genotyping of MIR196A2 (rs11614913) polymorphism was carried out by T‐ARMS‐PCR using a thermal cycler (Super Cycler, Model: SC300, Australia), as shown in Table 2. Online tool Primer 1 for rs11614913 was used for primer designing (as shown in Table 2), PCR reactions were performed in each PCR tube in 30 μL of total volume. The reaction mixture was composed of 18 μL of DreamTaq Green PCR Master Mix (2×) (Catalog number: K1081, Thermo Fisher Scientific, MA, USA), 5 μL of nuclease‐free water, 2 μL of template DNA, 1 μL of forward outer primer, 1 μL of reverse outer primer, 1.5 μL of reverse inner primer, and 1.5 μL of forward inner primer. The PCR conditions for initial denaturation were 95°C for 5 min, followed by 35 cycles including final denaturation at 95°C for 40 s, annealing temperature at 64°C for 30 s, initial extension at 72°C for 40 s, followed by final extension at 72°C for 10 min.

**TABLE 1:** The list of studied SNPs rs6505162 and rs11614913, their official name, mature miRNA sequences, chromosomal location, and MAF.

SNP ID	miRNA gene name	Name of mature miRNA sequences	Chromosome No.	miRNA location (coordinates)	Coded allele	Other allele	MAF
rs6505162	*MIR423*	hsa‐miR‐423‐5p	17	30,117,079–30,117,172 [+]	A	C/G/T	0.50
		hsa‐miR‐423‐3p					
rs11614913	*MIR196A2*	hsa‐miR‐196a‐5p	12	53,991,738–53,991,847 [+]	C	T	0.49
		hsa‐miR‐196a‐3p					

**TABLE 2:** List of primers (Outer and Inner primer) *of MIR196A2* (rs11614913) and *MIR423* (rs6505162) used in T‐ARMS PCR and for conventional PCR.

	SNP name	Primer sequences	PCR product size
T‐ARMS PCR	rs11614913‐F‐I (T)	5‐AGTTTTGAACTCGGCAACAAGAAACGGT‐3	199 bp
	rs11614913‐R‐I (C)	5‐GACGAAAACCGACTGATGTAACTCCGG‐3	153 bp
	rs11614913‐F‐O	5‐ACCCCCTTCCCTTCTCCTCCAGATAGAT‐3	297 bp
	rs11614913‐R‐O	5‐AAAGCAGGGTTCTCCAGACTTGTTCTGC‐3	
	rs6505162‐F‐I (A)	5‐TGAGGCCCCTCAGTCTTGCTTCCCAA‐3	228 bp
	rs6505162‐R‐I (C)	5‐CAAGCGGGGAGAAACTCAAGCGCGAGG‐3	160 bp
	rs6505162‐F‐O	5‐TTTTCCCGGATGGAAGCCCGAAGTTTGA‐3	336 bp
	rs6505162‐R‐O	5‐TTTTGCGGCAACGTATACCCCAATTTCC‐3	
Conventional PCR	rs11614913‐F	5‐CAGATGGACAAGACAGGGGA‐3	740 bp
	rs11614913‐R	5‐CCAGGCCACCTCTTACTCAA‐3	
	rs6505162‐F	5‐TGAGTGATCCGGGAGTTAGG‐3	713 bp
	rs6505162‐R	5‐CAAGTAGCCATGGGGCAAG‐3	

*Note*: The amplified product of conventional PCR was further processed for Sanger sequencing.

### 2.4 Genotyping of *MIR423* (rs6505162)

Similarly, genotyping of hsa‐miR‐423 (rs6505162) polymorphism was also carried out by T‐ARMS‐PCR. For rs6505162, PCR reactions were performed in PCR tubes in 25 μL of total volume. The reaction mixture was composed of 13 μL of DreamTaq Green PCR Master Mix (Thermo Fisher Scientific, MA, USA), 4 μL of nuclease‐free water, 2 μL of template DNA, 1 μL of forward outer primer, 1 μL of reverse outer primer, 1.5 μL of reverse inner primer, and 1.5 μL of forward inner primer. The PCR conditions were: initial denaturation was accomplished at 95°C for 4 min, followed by 35 cycles including denaturation at 95°C for 30 s, annealing temperature at 57°C for 35 s, extension at 72°C for 30 s, followed by a final extension at 72°C for 5 min. The amplified PCR products were separated on 2% agarose gel and visualized under a UV Trans illuminator (Meadowvale Way, Sparks NV, USA).

### 2.5 Sanger sequencing

To further validate the genotyping results of the T‐ARMS PCR, primers for Sanger sequencing were designed using an online tool (https://primer3.ut.ee/). The list of primers for the amplification of whole gene sequences of *MIR196A2* and *MIR423* including rs11614913 and rs6505162 using conventional PCR and Sanger sequencing is shown in Table 2 (Sanger et al., 1977).

### 2.6 Statistical analysis

Both MI patients and control genotypic data were checked for deviations from the Hardy–Weinberg equilibrium (HWE). Following several statistical models, including co‐dominant, homozygous dominant, homozygous recessive, heterozygous, and additive models, the relationship of each variable was assessed using Chi‐square and Fisher's exact test by calculating the odds ratio (OR) at 95 percent confidence intervals. The results were considered statistically significant at P ≤ 0.05.

## 3 RESULTS

### 3.1 Prevalence of studied subjects

A total of 400 MI patients and 336 healthy individuals were assessed in this study. The characteristics of the cases and healthy participants are listed in Table 3. In cases (of MI patients), the mean age was 60 (25–100), the mean blood pressure (B.P) was 148/96 (220/144–77/49), and the number of coronary artery disease (CAD) patients was 14, the number of hypertension (HTN) patients was 47, the number of diabetes mellitus patients was 35, the number of NSTEMI patients (Trop I > 0.03 ng/mL) was 68, and the number of STEMI patients was 332. In control samples, the mean age was 42 years.

**TABLE 3:** Pre‐clinical data about myocardial infarction, blood pressure, coronary artery disease, hypertension, and diabetes mellitus of the MI patients as well as healthy controls.

Categories	Mean age (year)	Gender	Mean B.P Systolic/diastolic (mmHg)	CAD	HTN	DM	Type of MI (trop I > 0.03 ng/ML)
Case (MI)	60 (25–95)	Male = 275	148/96 (220/144–77/49)	Yes = 14	Yes = 47	Yes = 35	NSTEMI = 68
		Female = 125		No = 386	No = 353	No = 365	STEMI = 332
Controls	42 years	Male = 210	Absent	Absent	Absent	Absent	Absent
		Female = 126					

Abbreviations: BP, blood pressure; CAD, coronary artery disease; HTN, hypertension; DM, diabetes mellitus; MI, myocardial infarction.

### 3.2 Genotyping of *MIR196A2* (rs11614913)

**FIGURE 1:**
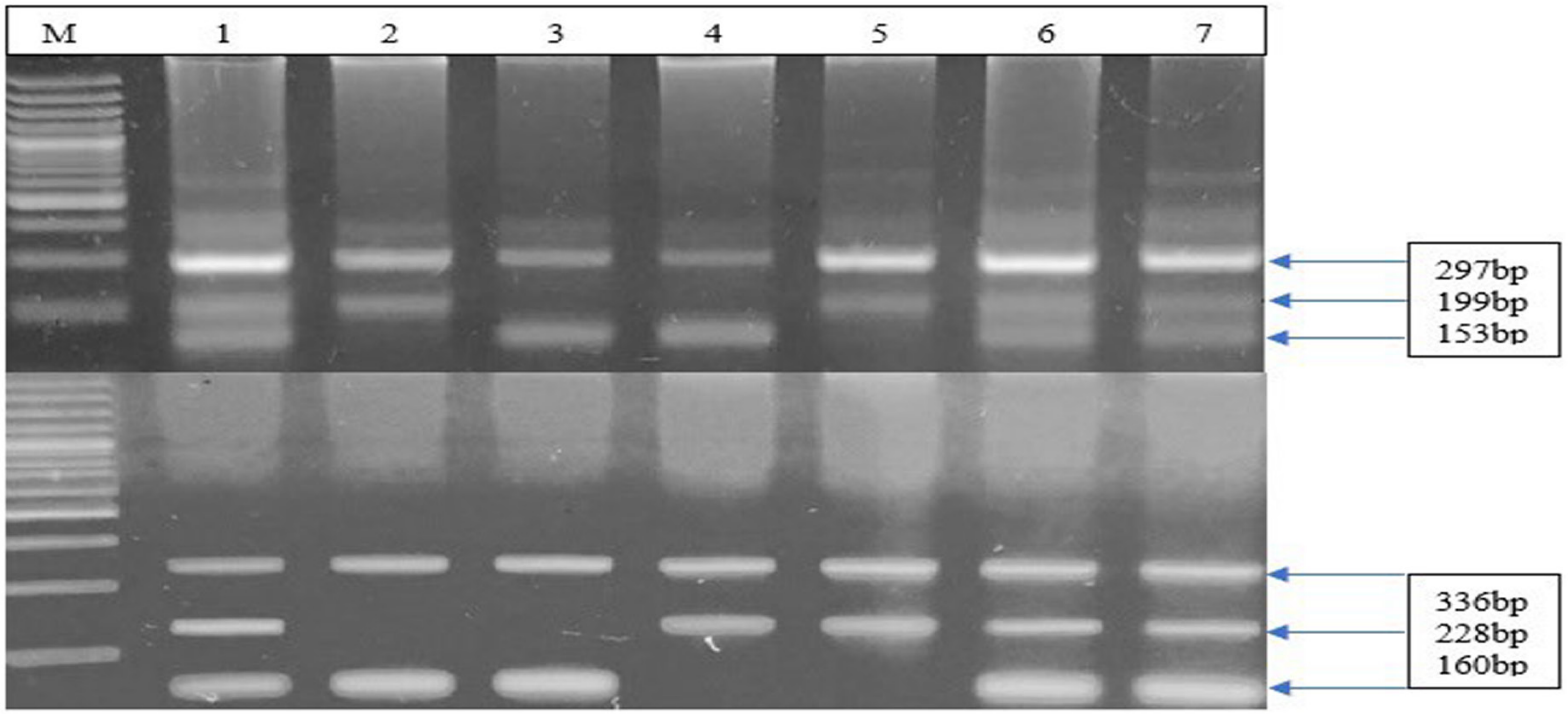
Representative electrophoresis gel picture of Tetra‐Primer Amplification Refractory Mutation System Polymerase Chain Reaction (T‐ARMS PCR) for the genotyping of SNP rs11614913 (upper) and rs6505162 (lower) in case and healthy control samples. M = Marker, lane = 1, 6 & 7 = CT, 2 & 5 = TT, and 3 & 4 = CC (upper) and M = Marker, 1, 6 & 7 = CA, 2 & 3 = CC, and 4 & 5 = AA (lower).

Through genotyping distribution, homozygous CC was found in 135, heterozygous CT in 223, and homozygous TT in 42 cases. CC was observed in 177, CT in 124, and TT in 35 control participants (*χ* ^2^ = 29.19, 2; *p* < 0.0001). Figure 1 shows a representative genotyping gel picture of rs6505162 and rs11614913. The frequency of the “C” allele was found at 493 in patients and 478 in controls. While allele “T” was found 307 in patients and 194 in controls. *p*‐value = 0.0001, OR = 0.65 (0.52 to 0.80). Homozygous dominant model analysis was assisted for the influence of the major allele on *MIR196A2* (rs11614913). A total of 135 of the patients and 177 of the controls had homozygous CC. Similarly, TT + TC was also found in 265 of the patient cases and 159 of the controls. The distribution of (rs11614913) was significant (OR = 0.45 (0.34 to 0.61), *p* < 0.0001). The lined red sentence is incomplete. Similarly, the influence of minor alleles on the *MIR196a2* relationship was investigated using recessive model analysis. Homozygous for “TT” was discovered in 42 cases and 35 controls. In cases, “CC + TC” was 358, whereas, in controls, it was 301 (OR = 1.009 (0.63 to 1.63), *p* > 0.999), as shown in Table 4.

**TABLE 4:** Statistical analysis for calculating the allelic and genotypic frequencies of rs11614913 and rs6505162 in MI patients compared to healthy individuals using inheritance models the co‐dominant, dominant, recessive, and additive models.

Gene (accession number)	Statistical models	Genotypes	Cases	Control	Odds ratiο (95% CI)	*χ* ^2^‐value, df	*p*value
*MIR196A2* (rs11614913)	Co‐dominant	CC	135	177	–	29.19, 2	<0.0001
		CT	223	124			
		TT	42	35			
	Dominant	CC	135	177	0.45 (0.34–0.61)	–	<0.0001
		CT + TT	265	159			
	Recessive	TT	42	35	1.009 (0.63–1.63)	–	>0.9999
		CT + CC	358	301			
	Additive	C	493	478	0.65 (0.52–0.80)	–	0.0001
		T	307	194			
*MIR423* (rs6505162)	Co‐dominant	CC	95	125	–	24.29, 2	<0.0001
		CA	241	140			
		AA	64	39			
	Dominant	CC	95	125	0.44 (0.32–0.61)	–	<0.0001
		AA + AC	305	179			
	Recessive	AA	64	39	1.29 (0.85–1.98)	–	0.28
		CC + AC	336	265			
	Additive	C	431	390	0.65 (0.52–0.81)	–	0.0001
		A	369	218			

### 3.3 *MIR423* (rs6505162) genotype distribution

**FIGURE 2:**
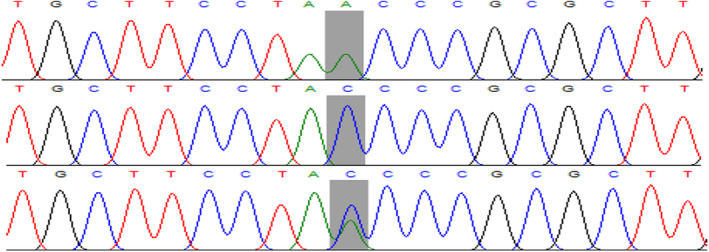
DNA sequencing confirmation for the homozygous and heterozygote genotypes of rs6505162 in the MIR423 gene.

Through genotyping distribution, homozygous CC was observed in 95, heterozygous CA in 241, and homozygous AA in 64 cases While CC was observed in 125, CA in 140, and AA in 39 in control participants (*χ* ^2^ = 24.29, 2, *p* < 0.0001). The allele “C” was present in 431 cases and 390 controls based on allele frequency distribution. While allele “A” was found in 369 of the cases and 218 of the controls. *p*‐value = 0.0001, OR = 0.65 (0.52 to 0.81). The major allele influence on *MIR423* (rs6505162) was assisted by homozygous dominant model analysis. In 95 cases and 125 controls, homozygous CC was found. Similarly, AA+AC had 305 cases and 179 controls. The distribution of (rs6505162) was significant (OR = 0.44 (0.32 to 0.61), (*p* < 0.0001)). Similarly, the influence of minor alleles on the *MIR423* relationship was investigated using recessive model analysis. Homozygous for “AA” was discovered in 64 cases and 39 controls. In cases, “CC + AC” was 336, whereas, in controls, it was 265 (OR = 1.29 (0.85 to 1.98), *p* = 0.28). Figure 2 illustrates the Sanger sequencing chromatograph confirming the T‐ARMS PCR genotyping results of rs6505162.

### 3.4 Association of *MIR196a2* and *MIR423* with myocardial infarction

Using different inheritance models (co‐dominant, additive, homozygous dominant, and homozygous recessive), the current study found that rs11614913 and rs6505162 are associated with the risk of MI. Different statistical models were used to evaluate rs11614913 and rs6505162. The rs11614913 genotypes showed significant association in co‐dominant [*χ* ^2^ = 29.19, 2; *p* < 0.0001], homozygous dominant [OR = 0.45 (0.34 to 0.61); *p* < 0.0001], and additive model [OR = 0.65 (0.52 to 0.80); *p* = 0.0001] while homozygous recessive model showed insignificant association [OR = 1.009 (0.63 to 1.63); *p* > 0.999]. Similarly, the rs6505162 genotypes showed significant association in co‐dominant [*χ* ^2^ = 24.29, 2; *p* < 0.0001], homozygous dominant [OR = 0.44(0.32 to 0.61); *p* ≤ 0.0001], and additive model [OR = 0.65 (0.52 to 0.81); *p* = 0.0001], but its association was insignificant at homozygous recessive model [OR = 1.29 (0.85 to 1.98); *p* = 0.28].

## 4 DISCUSSION

The most prevalent kind of CVD is myocardial infarction (MI), which is characterized by the necrosis of heart muscles due to ischemia (Lu et al., 2015) and is caused by the inheritance of many genetic variations that work along with environmental factors to enhance disease progression (Guella et al., 2009). The goal of this study was to look at the relationship between two microRNA SNPs (*miR‐196a2* rs11614913 and *miR‐423* rs6505162) with MI in a Pakistani population. The most intriguing and innovative mechanism in myocardial infarction, heart failure, and hypertrophy control is the expression of miRNAs within the myocardium (Bronze‐da‐Rocha, 2014). MicroRNA is an RNA interference (RNAi) regulatory system that inhibits protein translation by attaching to complementary regions at 3′ non‐translated regions in target messenger RNAs (Fu et al., 2015). Many studies have reported miRNAs as diagnostic indicators for diseases such as cancer (Lu et al., 2005), nervous system disease (Sheinerman et al., 2017), immunological disease (Carissimi et al., 2009), and cardiovascular disease (Ji et al., 2007) due to their excellent conservation and tissue specificity. This study investigated the relationship between MIR196A2 variant rs11614913 and MIR423 variant rs6505162 with susceptibility to MI in the Pakistani population. *MiR‐196a* deregulation is a common occurrence in cancer, indicating that it plays a key role in carcinogenesis and also has been linked to various critical biological processes, including development, cell differentiation, immunology, inflammation, and virus defense (Chen et al., 2011). The present study demonstrated that the SNP rs11614913 located in the microRNA‐196a2 gene was associated with an increased susceptibility to AMI within the Pakistani population. According to the study conducted by Wang et al. (2018), it was found that individuals carrying the TC, CC, or TC + CC miRNA‐196a2 genotypes in a Chinese population exhibit a higher susceptibility to the development of CAD. The findings of Zhi et al. (2012) research indicate that the presence of the miR‐196a2 rsl11614913 C allele in individuals with CHD is associated with an elevated susceptibility to AMI and other severe cardiovascular complications within the southeastern regions of China. Osmak et al. (2018) also reported the same results in the ethnic Russian population: Patients with myocardial infarction had a higher frequency of allele C in polymorphism rs11614913 (MIR196A2) compared to the control group. Additionally, the TT genotype was significantly lower in the MI group compared to the control group (Osmak et al., 2018). Another study conducted within the Greek population revealed that the MiR196a2 C > T and miR499 A > G polymorphisms were significantly associated with elevated risk of CAD and MI (Agiannitopoulos et al., 2021). In contrast to our study's findings and the studies mentioned above, certain investigations have reported that the genetic variant MIR196a2 (rs11614913) does not play a significant role in the development of MI. The study conducted by Wang et al. (2017) found no significant association between the MiR‐196a2 rs11614913 polymorphism and the risk of CAD in the Asian population (Wang et al., 2017). A study by Mahmoud et al. found that individuals with the TT genotype and T allele in miR‐196a2 (rs11614913) have a lower risk of developing ischemic stroke. The study showed that the frequency of the TT genotype was significantly lower in stroke patients in Egypt compared to the control group (Mahmoud et al., 2020).

The variant (rs6505162) in miR423 has been found to be associated with a variety of disorders including breast cancer (Morales et al., 2016), esophageal cancer (Wang et al., 2013), primary ovarian syndrome (Rah et al., 2015), and rheumatoid arthritis (Ullah et al., 2022).

The present case–control study also investigated the potential relationship between miRNA*423* rs6505162 polymorphisms and MI susceptibility in a Pakistani population. Our results showed that *MIR423* rs6505162 polymorphisms are significantly associated with the MI risk. Previous research indicated that the microRNA‐423 CA genotype and A allele are associated with a higher risk of coronary artery disease in the Indian population (Jha et al., 2019). In a 2013 study by Olivieri et al., individuals diagnosed with NSTEMI were found to have increased levels of circulating miR‐21 and miR‐423‐5p (Olivieri et al., 2013). A recent study conducted in 2022 reported a significant association between MIR423 and individuals diagnosed with STEMI (Cecconi et al., 2022). A previous study reported that circulating miR‐423‐5p may serve as an early marker for myocardial infarction in Poland. The study observed that the concentration of miR‐423‐5p in plasma is significantly elevated during the early stages of AMI but returns to normal within 360 min (Nabiałek et al., 2013). Contrary to our research findings, some studies have reported that MIR423 is not associated with MI. Xue et al. (2019) found no significant difference in the level of miR‐423 between patients with AMI and control subjects (Xue et al., 2019). In a study conducted by Bauter and colleagues, it was found that the levels of miR‐423‐5p were not a dependable biomarker for assessing left ventricular remodeling following myocardial infarction (Bauters et al., 2013). A previous study has revealed that miR‐423‐5p lacks the requisite diagnostic accuracy for heart failure. However, when utilized in conjunction with NT‐proBNP, the diagnostic accuracy is significantly enhanced (Ellis et al., 2013).

## 5 CONCLUSION

The present study has determined a significant association between the MIR196A2 variant rs11614913 and the MIR423 variant rs6505162 with the risk of myocardial infarction in the Pakistani population. However, some concerns need to be addressed regarding the current study. Firstly, it is worth noting that the case–control study was carried out in a hospital setting, which may not accurately reflect the wider population. Secondly, increasing the sample size to enhance statistical power may be beneficial.

## AUTHOR CONTRIBUTIONS

Muhammad Uzair and Yasir Ali analyzed the data and participated in manuscript writing. Muhammad Uzair, Taqweem Ul Haq, Sajjad Ali and Manzar Hussain collected blood samples and clinical data. Muhammad Uzair, Taqweem Ul Haq and Manzar Hussain performed the laboratory experiments. Aftab Ali Shah and Fazal Jalil supervised and helped in writing and editing the manuscript. Fazal Jalil, Yasir Ali and Aftab Ali Shah edited the manuscript. All authors read and approved the final manuscript.

## FUNDING INFORMATION

The author(s) received no specific funding for this work.

## CONFLICT OF INTEREST STATEMENT

The authors declare that they have no conflicts of interest.

## STUDY APPROVAL STATEMENT

The study protocol was reviewed and approved by the Advanced Study and Research Board (ASRB), University of Malakand, in its 67th meeting held on November 16, 2021.

## CONSENT TO PARTICIPATE STATEMENT

The study's objective was explained in the local language to potential participants to obtain informed consent, and only those who provided consent were included in the research sample.

## Data Availability

The data that support the findings of this study are available from the corresponding author upon reasonable request.
